# In Silico Design and Subsequent Expression of Human Papillomavirus-16 and -18 L1 Vaccine Antigens in Broccoli

**DOI:** 10.3390/vaccines14030261

**Published:** 2026-03-13

**Authors:** Neelam Batool, Khadeeja Ahsan, Kainat Qadeer, Al Fajar, Alveena Farid, Muhammad Sameeullah, Fatima Ijaz, Muhammad Suleman Malik, Fizza Ahmad Tariq, Andreas Günter Lössl, Martin Müller, Mohammad Tahir Waheed

**Affiliations:** 1 Department of Biochemistry, Faculty of Biological Sciences, Quaid-i-Azam University, Islamabad 45320, Pakistan; neelamchaudry93@gmail.com (N.B.); khadeejaahsan99@gmail.com (K.A.); kainatqadeer00@gmail.com (K.Q.); fajr9800@gmail.com (A.F.); fatima.ijaz.a@gmail.com (F.I.); sulemanmalik.04@gmail.com (M.S.M.); fizza2297@gmail.com (F.A.T.); 2 Sharif Medical City Hospital, Lahore 54000, Pakistan; alveenafarid24@gmail.com; 3 Department of Field Crops, Faculty of Agriculture, Bolu Abant Izzet Baysal University, Bolu 14030, Türkiye; sameepbg@gmail.com; 4 Centre for Innovative Food Technologies Development, Application and Research, Bolu Abant Izzet Baysal University, Bolu 14030, Türkiye; 5 Department of Business and Management, IU International University of Applied Sciences, Berg-am-Laim-Str. 43, 81673 Munich, Germany; andreas.loessl@iu.org; 6 German Cancer Research Center, Im Neuenheimer Feld 242, 69120 Heidelberg, Germany; martin.mueller@dkfz-heidelberg.de

**Keywords:** cervical cancer, *Agrobacterium*-mediated transformation, HPV, Gateway^®^ cloning, immunoinformatics, LTB-L1

## Abstract

**Background**: Cervical carcinoma remains a widespread cancer worldwide, primarily caused by persistent infection with high-risk human papillomavirus (HPV). HPV types 16 and 18 account for approximately 70% of cervical cancer cases. Although prophylactic HPV vaccines are commercially available, their high cost and reliance on expensive expression platforms limit their accessibility in developing countries. **Objectives**: This study aimed to develop a cost-effective, plant-based HPV vaccine candidate by expressing capsomeric HPV-16 and HPV-18 L1 antigens in *Brassica oleracea* (broccoli). **Methods**: Modified L1 from HPV types 16 and 18 were designed to retain capsomeric assembly and fused with heat-labile enterotoxin B subunit (LTB). Immunoinformatics analyses were used to assess antigenicity, epitope distribution, and structural characteristics. Codon-optimized genes were cloned using Gateway^®^ technology and expressed in broccoli via *Agrobacterium*-mediated transformation. Transgenic plants were validated by PCR and qRT-PCR. Protein accumulation was quantified, and immunogenicity was evaluated in mice. **Results**: PCR and qRT-PCR confirmed the stable integration of two copies of the *LTB-L1* transgenes in broccoli plants. Western blotting detected L1 protein at ~56.5 kDa, indicating the cleavage of the LTB-L1 fusion protein. The correct folding of L1 capsomeres was verified by antigen-capture ELISA. The recombinant proteins accumulated to approximately 0.33% and 0.35% of total soluble protein for HPV-16 and HPV-18, respectively. The immunization of mice with transgenic L1 induced significant humoral immune responses, comparable to those elicited by purified VLPs. **Conclusions**: The results demonstrate broccoli as a promising platform for the expression of immunogenic HPV L1 capsomeres and highlight its potential for the development of affordable, plant-based HPV vaccines.

## 1. Introduction

The fourth-most widespread type of cancer in women is cervical cancer. Globally, 661,021 women were diagnosed with cervical cancer in 2022 [1]. It is a major contributor to women’s cancer-related mortality, accounting for around 350,000 fatalities worldwide in a single year [2]. Approximately 94% of the deaths from cervical cancer are in underdeveloped and low-income countries [3]. According to projections by the World Health Organization (WHO), the worldwide incidence of cervical cancer is anticipated to increase by 16.9%, with a corresponding 21.1% rise in mortality, by the year 2030. These alarming trends highlight the critical need for innovative strategies and improved vaccination efforts to control HPV infection [2]. In over 99% of instances, the continuing infection of carcinogenic human papillomavirus (HPV) strains has been identified as the primary contributor to the onset of cervical cancer.

Globally, HPV remains the most widespread sexually transmitted infection [4]. Nearly 200 HPV types exist, out of which at least 14 are recognized as the high-risk types involved in oncogenesis. Among these 14 types, HPV-16 and -18 are the most prevalent types of human papillomavirus, responsible for 70% of invasive cervical cancer and precancerous cervical lesions [5]. L1, the major capsid protein in human papillomavirus, is a 55 kDa protein that has the intrinsic ability to self-assemble into VLPs spontaneously with the help of disulphide cross-linkages between neighbouring L1 molecules, forming an icosahedral surface lattice [6]. Cys175 in the invaded capsomere’s L1 and the Cys428 in the invading L1’s C-terminus contribute to the intercapsomeric disulphide bonding.

These two cysteines are highly conserved among all HPV-related L1 proteins [7]. If these two highly conserved cysteines are replaced by serine residues, the L1 proteins are not able to assemble into VLPs and remain in the form of individual capsomeres, which are pentameric units [8]. Assembled VLPs possess potent immunogenic traits due to their innate mechanism of B-cell recognition of the characteristic spacing of surface epitopes on the regular icosahedral surface of a mature virion [9]. VLPs are highly immunogenic entities, as they can induce notable levels of neutralizing antibodies and have been shown to induce immunity to HPV infections [10,11].

Three VLP-based vaccines have been introduced commercially, out of which one is the tetravalent vaccine Gardasil-4 (by Merck, Rahway, NJ, USA), obtained from a yeast expression system covering HPV types 16-, 18-, 6- and 11-based infections, and another is a bivalent vaccine Cervarix (by GlaxoSmithKline, London, UK) that specifically covers only HPV types 16 and 18 [11,12,13]. An updated form of Gardasil 4 that has efficacy against nine HPV types, referred to as Gardasil 9, is an efficient vaccine to counter HPV infections. The nine HPV types are 6, 11, 16, 18, 31, 33, 45, 52 and 58; hence, Gardasil 9 (by Merck & Co.) can help to prevent 90% of cervical cancer cases caused by the aforementioned high-risk HPV types [14]. In addition, three newly developed prophylactic HPV vaccines—Cecolin^®^, Walrinvax^®^, and Cervavac^®^—have received license in two countries: China and India. The World Health Organization (WHO) has prequalified both Cecolin^®^ and Walrinvax^®^ as of 2024 [2]. Cecolin^®^, a Chinese bivalent vaccine, produced by Xiamen Innovax Biotech using an *Escherichia coli* expression system, contains 40 µg of HPV-16 and 20 µg of HPV-18 recombinant L1 virus-like particles (VLPs). Similarly, another bivalent VLP-derived vaccination that targets HPV strains 16 and 18 is called Walrinvax^®^. It is made with *Pichia pastoris* as the expression host.

Cervavac^®^, developed by the Serum Institute of India, is a quadrivalent vaccine, targeting HPV types 6, 11, 16, and 18 [14]. These vaccines, although highly effective, are very expensive because of their complicated production processes, cold-chain storage and sterile intramuscular delivery. Despite the fact that more than 8/10 cases of cervical cancer occur in low-income countries, the currently available vaccines based on HPV L1 VLPs are prohibitively expensive in many high-burden countries (e.g., around $4–5 per dose, even under Gavi pricing, with delivery-related costs adding significantly). This explains why second-generation vaccines should be developed with a low cost. In addition, current efforts toward a global rollout of HPV vaccination, such as Gardasil 9 programs, are subject to the same implementation barriers faced by low- and middle-income countries, characterized by supply limitations, as well as cold-chain and programmatic costs, which all together hamper the swift scale-up of coverage [1,15]. Such chronic obstacles highlight the necessity of different vaccine manufacturing systems that should be low-cost and scalable, and can be used alongside simplified distribution plans.

The plant-based expression of L1 capsomeres can reduce manufacturing complexity and expense, offer enhanced thermostability (e.g., lyophilization, no cold chain), and thus represent a promising affordable alternative to VLPs for low-resource settings, supporting the urgent need for scalable, safe, and economical HPV vaccines [14]. An in silico analysis of HPV target genes for prophylactic vaccine design provides a logical and systematic approach to guide experimental research, reducing dependence on trial-and-error experimentation and streamlining vaccine development by focusing on the most promising candidates [16].

Bioinformatics offers a great opportunity to predict the immunological and physiochemical properties of a vaccine construct, along with its safety and efficacy, thereby enabling researchers to design a safe and potent vaccine. The latest advancement in bioinformatics has provided this field with a large number of vast and significantly improved methods for vaccine design [17]. In this regard, multiple approaches are now available, such as Reverse Vaccinology (RV) and structural vaccinology, that boost the overall procedure of vaccine design by a significant rate [18]. Moreover, the latest development of a safe and efficient Multi-Epitope Subunit Vaccine (MESV) requires the determination of immunogenic, non-toxic, and safe epitopes that can generate strong cell-mediated and humoral immunity [19]. Consequently, updated online servers have been introduced for the accurate prediction of immune-dominant epitopes [20].

Plants have also become an attractive alternative platform for recombinant vaccines, therapeutic proteins and biopharmaceutical production. Several vaccines made of plants have shown good results in clinical and preclinical trials, and a plant-made enzyme, glucocerebrosidase, in the management of Gaucher’s disease is already on the market [21,22]. Many studies have revealed that plants are capable of producing antigens for various pathogens in high concentrations without the loss of original conformation, which allows them to induce protective immune responses after oral administration [23]. A plant-based system has various benefits such as being inexpensive to produce, having the ability to be scaled, minimal biosafety issues, and the possibility to be grown locally. Notably, it minimizes the use of cold-chain infrastructure, as well as mitigating transportation-related costs [24,25]. Edible plants specifically gained interest as potential hosts to develop oral vaccines since they provide the ability to bio-encapsulate antigens into plant cells, retain proteins to prevent degradation in the gastrointestinal tract, and allow delivery without needles. Some edible crops, such as potato, tomato, rice, maize, and lettuce, have been used in experiments for the expression of vaccine antigens and therapeutic proteins, and they have proven successful in the application of plant-based oral immunization strategies [26,27,28]. Edible crops are also better in their potential usage in raw or partially processed form, thus evading purification-related costs.

*Brassica oleracea* var. *italica* (broccoli) can be an attractive plant species for the expression of therapeutic proteins and vaccines due to its edible nature. It shows a comparatively high yield of biomass, possesses established *Agrobacterium*-mediated transformation and regeneration procedures, and is broadly used in minimally processed form across the world. There are very few reports of using broccoli as an expression platform for vaccine antigens [25]. Furthermore, broccoli has small edible florets that are easy to dose and store, which makes this crop ideal in the production of oral vaccines at large scale. These characteristics make broccoli a feasible and strategically favourable substitute to the conventionally used plants in the production of affordable vaccines.

The present study aimed to express modified L1 antigens from HPV types 16 and 18, fused with *Escherichia coli* heat-labile enterotoxin subunit B (LTB) as adjuvant, in broccoli. Vaccine constructs of LTB-L1 (for both HPV types: 16 and 18) were created using the modified *L1* gene’s sequence. Following the generation of vaccine constructs, a hierarchical approach was adopted that involved physiochemical analysis, immunogenicity, allergenicity, and designed structures’ solubility prediction. Thereafter, a structural model of the vaccine constructs was predicted, refined, and subsequently validated. Final transformation vectors were constructed using the Gateway^®^ cloning of expression cassettes in a pGWB5 vector. *Agrobacterium*-mediated transformation in *Brassica oleracea* (Broccoli) var. *italica*. cultivar Marathon was used to express the target genes. Conventional PCR was used to validate transgene integration, and quantitative real-time PCR (qRT-PCR) was employed to determine the transgene copy number. Western Blotting was used to verify the expression of transgenes, and ELISA was used to verify that the expressed L1 antigen’s structural epitopes were correctly assembled. Finally, the immunogenicity of the expressed antigens was investigated in mice.

## 2. Materials and Methods

### 2.1. Retrieval of Gene Sequences, Modification and Vaccine Constructs

The L1 protein sequence of HPV-16, named L1-WT16, consisting of 505 amino acids (Gene bank accession no. KM058645.1), was modified by the deletion of the first 10 N-terminus amino acids. The addition of methionine with leucine in the start of sequence, along with the substitution of two cysteine residues to serine at positions 175 and 428 and aspartic acid at position 202 to histidine leads to the formation of L1-Cap16 of 497 amino acids. For HPV-18 L1 protein sequence, named L1-WT18, consisting of 507 amino acids (Gene bank accession no. MF288717.1), ten amino acid residues were deleted N terminally. Moreover, the addition of methionine with leucine at the start of sequence and two cysteine-to-serine substitutions were performed at positions 175 and 429, which led to L1-Cap18 of 499 amino acids. These modifications were done to retain the L1 conformation to capsomeric assembly, rather than the virus-like particles (VLPs) formation.

In L1-Cap16 and L1-Cap18 of HPV-16 and 18, respectively, the protein sequence of *E. coli* LTB (ETEC, Genbank accession number M17874) was integrated at the N-terminus as an adjuvant for eliciting enhanced immunogenicity, along with a separator sequence to separate adjuvant sequence from the subsequent modified L1 amino acid sequence. Finally, at the C-terminus, six histidine residue sequences were inserted for both HPV-16 and -18 constructs. This yielded LTB-L1-Cap16-His (657 amino acids) and LTB-L1-Cap18-His (659 amino acids), termed as HPV-16 LTB-L1 vaccine construct and HPV-18 LTB-L1 vaccine construct, respectively.

### 2.2. Epitope Prediction

In this study, immunodominant epitopes were predicted specifically for the activation of B-cells, Cytotoxic T-lymphocytes (CTLs), and Helper T cells (HTLs) by different online tools in the HPV-16 LTB-L1 vaccine construct and HPV-18 LTB-L1 vaccine construct for their competency for generating cell-mediated and humoral immunity. For the activation of CTLs, an Immune Epitope Database (IEDB) tool (http://tools.immuneepitope.org/mhci/, accessed on 3 June 2021) MHC-I binding prediction software, using the recommended NetMHCpan EL 4.1 method, was employed. This analysis was focused on human HLA alleles, and the sequences (HPV-16 LTB-L1 and HPV-18 LTB-L1) were submitted for MHC-I epitope mapping. Further, immunodominant epitopes for Helper T cells (HTLs) were computationally predicted through the MHC-II tool provided by IEDB (http://tools.iedb.org/mhcii/, accessed on 18 June 2021). The in silico analysis considered MHC-II binding epitopes, with parameters set to default conditions, and sequences (HPV-16 LTB-L1 and HPV-18 LTB-L1) were submitted for epitope mapping. To predict B-cell epitopes in the vaccine constructs (both HPV-16 LTB-L1 and HPV-18 LTB-L1), the ABCpred tool (http://crdd.osdd.net/raghava/abcpred/, accessed on 30 June 2021) was used, with a selected threshold of 0.8, window length of 16, and no overlapping filter [29,30]. This integrated strategy sought to investigate the elicitation of a comprehensive immune response for vaccine constructs (HPV-16 LTB-L1 and HPV-18 LTB-L1). A variety of online programs, including ToxinPred, VaxiJen v2.0, and AllerTOP v2.0, were used to examine each epitope for toxicity, antigenicity, and allergenicity.

### 2.3. Characteristic Evaluation for Vaccine Constructs

Various parameters of physiochemical properties, including aliphatic index, molecular weight, atomic composition, instability index, amino acid composition, expected half-life under in vitro and in vivo conditions, extinction coefficient, theoretical isoelectric point (pI), and Grand Average of Hydropathicity (GRAVY) of both multiepitope HPV-16 and HPV-18 LTB-L1 vaccine constructs, were analyzed by utilizing the Expasy Protparam web server (https://web.expasy.org/protparam/, accessed on 12 July 2021). Further, the solubility of multiepitope vaccine constructs upon overexpression were assessed using the Scratch Protein Predictor tool (http://scratch.proteomics.ics.uci.edu/, accessed on 17 July 2021). Several bioinformatic tools—ToxinPred (http://crdd.osdd.net/raghava/toxinpred/, accessed on 19 July 2022), AllerTOP v. 2.0 (https://www.ddg-pharmfac.net/AllerTOP/, accessed on 19 July 2021) and VaxiJen v2.0 (http://www.ddg-pharmfac.net/vaxijen/, accessed on 21 July 2022)—were applied for evaluating the toxicity, allergenicity, and antigenicity of both HPV-16 and HPV-18 LTB-L1 vaccine constructs. In order to guarantee the uniqueness of the vaccination candidate, homology analysis was carried out using BLASTP (https://www.ncbi.nlm.nih.gov/blast/, accessed on 21 July 2021), with an E-value threshold of 0.05, aiming to identify any potential similarity with human proteins that could lead to adverse immunogenicity.

### 2.4. 3D Structure Generation and Validation

HPV-16 LTB-L1 and HPV-18 LTB-L1 vaccine constructs underwent a procedure for predicting the tertiary structure through the automated web-based server SWISS-MODEL (https://swissmodel.expasy.org/, accessed on 11 August 2021). This server is highly efficient for automated 3D structure generation from provided protein sequences along with quality functional assessment.

Following the submission of LTB-L1 vaccine construct sequences in FASTA format, the structure with the highest sequence coverage and the best overall quality metrics was selected among the models generated by SWISS-MODEL. The generated models were validated using the QMEAN (Qualitative Model Energy Analysis) z-scoring function and through Ramachandran plots; along with this, the Saves v6.0 server (https://saves.mbi.ucla.edu/, accessed on 15 August 2021) was also used for ERRAT assessment. PyMOL (https://pymol.org/, accessed on 18 August 2021) was used to visualize the completed 3D constructions.

### 2.5. Discontinuous Mapping of B-Cell Epitopes

The three-dimensional discontinuous epitopes recognized by B-cells, considered crucial for inducing antibody production, were mapped for multiepitope vaccine candidates with the help of a webserver named ElliPro (http://tools.iedb.org/ellipro/, accessed on 31 August 2021). ElliPro utilized PDB files of 3D models for epitope computation, with default parameters adjusted to the values of 0.5 and 6 for ‘Maximum score’ and ‘Maximum distance’, respectively.

### 2.6. Codon Optimization and Gateway^®^ Cloning

**Figure 1:**
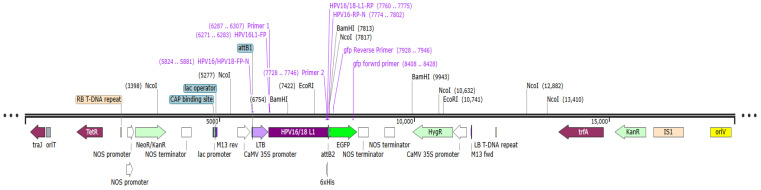
**Schematic illustration of pEXP-HPV-16 and -18 binary vectors depicting LTB-L1 expression cassette**. The vector map illustrates the expression cassette containing the *L1* gene within the pDEST-pGWB5 backbone. Additionally, the key elements of the said vector include traJ (oriT-recognizing protein); tetR (tetracycline resistance regulator); RB (right border repeat from nopaline C58 T-DNA); NOS promoter and NOS terminator (derived from nopaline synthase); NeoR/KanR (kanamycin resistance gene); CAP binding site (*E. coli* catabolite activator protein); lac promoter; lac operator; CaMV 35S promoter (strong constitutive promoter from cauliflower mosaic virus); attB1 and attB2 (Gateway^®^ recombination sites); LTB adjuvant; *L1* gene (HPV-16 and -18); 6x histidine tag; GFP (green fluorescent protein); HygR (hygromycin resistance gene); LB (left border repeat); trfA (trans-acting replication protein); IS1 (insertion sequence 1); and OriV (origin of replication).

The GenSmartTM Codon Optimization tool was employed to optimize the gene sequence according to the plant expression platform for expressing the *LTB-L1* gene in broccoli. Both HPV-16 LTB-L1 and HPV-18 LTB-L1 vaccine constructs were subsequently integrated in the pUC57 vector. The designed vectors were synthesized from BIOMATIK (Kitchener, ON, Canada). The final binary expression vectors, expressing the target *LTB-L1* gene, were created through the Gateway^®^ cloning approach. To clone *LTB-L1* gene from HPV types 16 and 18 in pDONR^TM^221, a BP recombination reaction was performed, resulting in intermediate entry vectors pENTR-HPV-16_Agr_ and pENTR-HPV-18_Agr_, respectively. The LR recombination reaction among entry vectors and destination vector pGWB5 led to the construction of final expression vectors pEXP-HPV-16_Agr_ and pEXP-HPV-18_Agr_. The expression of target *LTB-L1* gene in the final expression vectors was regulated by a strong constitutive cauliflower mosaic virus 35S (CaMV 35S) promoter and the nopaline synthase (nos) terminator. The final expression vectors pEXP-HPV-16_Agr_ and pEXP-HPV-18_Agr_ are shown in Figure 1. The Gateway^®^ BP and LR cloning procedures were performed in accordance with the manufacturer’s instructions [31]. The BP Clonase^®^ and LR Clonase^®^ kits were purchased from Invitrogen (Carlsbad, CA, USA). PCR using various sets of primers in the final expression vector confirmed that the *LTB-L1* gene was properly integrated at each stage of cloning.

### 2.7. Agrobacterium-Mediated Transformation and Plant Regeneration

The final transformation vectors pEXP-HPV-16_Agr_ and pEXP-HPV-18_Agr_ were transformed in *Agrobacterium tumefaciens* strain GV3101 via electroporation and subsequently utilized for the transformation of *Brassica oleracea* var. *italica* cultivar Marathon. The main inoculum of transformed GV3101 *Agrobacterium* strain was cultured in liquid LB medium containing supplementary antibiotics (50 mg/L kanamycin and rifamycin). The inoculated LB medium was kept overnight in a shaking incubator at 250 rpm and 28 °C. Following the overnight incubation, the inoculum with an OD_600_ value from 0.6 to 0.8 was centrifuged for 20 min at 5500× *g* and room temperature. The resulting bacterial pellet was suspended in liquid MS medium, with 200 μM acetosyringone added for efficient downstream infection.

In the present research, *Brassica oleracea* var. *italica* cultivar Marathon seeds were purchased from a local supplier. Seeds were sterilized to eliminate the presence of any surface-borne harmful microbes that otherwise would not only cause contamination during plant culturing but also influence the seed germination. The seeds were first treated with a 0.1% mercuric chloride (HgCl_2_) solution for a minute and subsequently washed three times using distilled water. Sterilized broccoli seeds were allowed to germinate at 25 °C on agar half-MS medium. Broccoli plants grown in vitro for 3–4 weeks were used for nodal explant preparation, with each nodal segment 5–8 cm in length. The excised explants were infected with *Agrobacterium* suspension for approximately 8 min. The infected nodal explants were kept in darkness for two days on regeneration media of plant (RMOP) supplemented with 20 g/L sucrose, 4.4 g/L MS salts, 100 mg/L myo-inositol, 0.1 mg/L NAA, 1 mg/L thiamine HCL and 1 mg/L 6-BAP. Further, 75 mg/L of kanamycin was utilized as the selection medium. In a growth environment, the *Agrobacterium*-mediated transgenic broccoli plants were cultivated inside a growth room at 25 ± 2 °C in ideal 16/8 h of light–dark conditions. The grown transformed broccoli plants after acclimatization were transferred to a greenhouse.

### 2.8. Molecular Confirmation of Transgene Integration

Conventional PCR was performed to confirm the presence of *LTB-L1* within transformed broccoli plants. As outlined by Muray and Thompson [32], genomic DNA was isolated from wild-type (WT) and potentially transgenic broccoli plants using 100 mg of leaves and the cetyltrimethylammonium bromide (CTAB) technique. In short, 10 Mm deoxyribonucleotide triphosphate (dNTPs), 1.5 mM MgCl_2_, 100 ng of template DNA, 1x Taq Buffer, 1.25 U of Taq polymerase (ThermoFisher Scientific, Waltham, MA, USA), and 1 µM primers were all included in the final volume of 25 µL PCR master mix. The first pair of primers, sense primer HPV-16/-18_IP_F (5′-ATGAATAAAGTAAAATTTTATGTTTTATT-3′) and anti-sense primer HPV-16/-18_IP_R (5′-GTTTTCCATACTGATTGC-3′), were employed to validate *LTB-L1* gene integration in the transformed broccoli nuclear genome. The first primer set’s annealing temperature was 49 °C. The second pair were sense primer HPV-16/18_NP_F (5′-GGGGACAAGTTTGTACAAAAAAGCAGGCTATATGAATAAAGTAAAATTTTATGTTTTATTTA-3′) and the anti-sense primer for HPV-16_NP_R (5′- GGGGACCACTTTGTACAAGAAAGCTGGGTACTA-3′) or the anti-sense primer of HPV-18_NP_R (5′-GGGGACCACTTTGTACAAGAAAGCTGGGTTTA-3′). The second primer set’s annealing temperature was 62.8 °C.

The frequency of *LTB-L1* gene insertion in the broccoli nuclear DNA was assessed through real-time PCR (RT-PCR) amplification using aforementioned *LTB-L1* specific primers on MyGo Pro Real-Time PCR Detection System (Stokesley Middlesbrough, UK). According to the method by Wen et al. (2012) [33], transgene copy number was determined through the standard curve method of relative quantification. This method involved the formation of serial dilutions of DNA extracted from transformed and wild-type broccoli for generating a standard curve against target (*LTB-L1*), as well as a reference (*β-actin*) gene. A reaction mixture was prepared comprising 100 ng extracted phytogenomic DNA, 1 µM *L1* specific primers, and 10 µL SYBR Green dye, with the final reaction volume being adjusted to 20 µL using nuclease-free water. The protocol was set in the MyGo Pro 3.2 Software before each quantitative real-time PCR run. The initial denaturation was done at 95 °C for 10 min, followed by a series of 45 cycles of three-step amplification, consisting of denaturation at 95 °C for 15 s, annealing at 49 °C for 60 s and extension at 72 °C for 20 s. The amplification of *LTB-L1* gene from both of the HPV types was done by using sense primer HPV-16/-18_FP (5′-ATGAATAAAGTAAAATTTTATGTTTTATT-3′) and anti-sense primer HPV-16/-18_RP (5′-GTTTTCCATACTGATTGC-3′), and, for *β*-actin gene, sense primer (5′-AGGTGCCCTGAGGTCTTGTTCC-3′) and anti-sense primer (5′-ATCAGCAATACCAGGGAACATAGT-3′) were utilized. The DNA samples isolated from the transformed and wild-type broccoli were serially diluted at 1, 10, 100 and 1000 ng for every reaction to establish a linear relationship between fluorescence level and DNA amount. The copy number of integrated transgenes *LTB-L1* in transgenic broccoli plants was calculated using the following formula:

$$\delta rline=rline{\left[{\left(\delta {SQ}_{trans}/{SQ}_{trans}\right)}^{2}+{\left(\delta {SQ}_{end}/{SQ}_{end}\right)}^{2}\right]}^{1/2}$$

### 2.9. Protein Expression and Detection

The seven-to-ten-days-grown transgenic and wild-type young broccoli leaves were subjected to total soluble protein (TSP) isolation using homogenization buffer (100 mM sodium chloride (NaCl), 2 mM PMSF, 10 mM EDTA pH 8, 14 mM b-mercaptoethanol 0.05% vol/vol Tween-20, 200-mM Tris–HCl pH 8, 0.1% wt/vol SDS, and 200 mM sucrose). Then, 100 mg of young leaves was frozen in liquid nitrogen and subsequently ground in a pre-chilled pestle and mortar. The buffer was added to finely powdered ground sample, followed by centrifugation at 14,000 rpm and 4 °C for 15 min. After the completion of centrifugation, the supernatant, having a pure TSP fraction, was stored at –20 °C to prevent protein degradation, until performing the downstream applications. Additionally, the isolated soluble protein was quantified through the gold standard colorimetric Bradford assay.

Further, the quantified protein extracts were pre-heated at 95 °C for 10 min with 4× SDS loading buffer. The samples were then loaded on to the wells of 4–10% NuPAGE^®^ Bis Tris Gel (Thermofisher, Waltham, MA, USA) in an XCell4 SurelockTM Midi Cell (Invitrogen, Carlsbad, CA, USA) to be electrophoresed employing the SDS-PAGE technique. The VLPs generated from baculoviruses (obtained from German Cancer Research Centre (DKFZ), Heidelberg, Germany) served as a positive control in western blotting. After electrophoresis, the gel containing resolved proteins was placed in a semi-dry transfer apparatus for transferring proteins onto a nitrocellulose membrane (ThermoFisher Scientific, Waltham, MA, USA). The blotted nitrocellulose membrane was dipped in a blocking buffer comprising 5% skim milk in Tris-buffered saline and 0.1% Tween-20 (TBST) for one hour at room temperature to reduce the non-specific background of primary antibody. Following the blocking step, the blot was washed thrice using PBS containing 0.1% Tween-20 (PBS-T) buffer for 10 min to remove excessive and unbound proteins in blocking buffer. The blot was then incubated overnight with the MD2H11 (obtained from German Cancer Research Centre (DKFZ), Heidelberg, Germany) primary monoclonal antibody specific to the L1 antigen, diluted with 1:10,000 PBS-T containing 0.05% sodium azide and 5% BSA. On the following day, the blot was washed and treated with secondary antibodies, specifically goat anti-mouse immunoglobulin (IgG) (GAMPO) coupled with horseradish peroxidase (HRP) (1:10,000 dilution in TBS-T with 5% BSA), for one and a half hours at room temperature. The blot was visualized in a chemiluminescence system (Fluor Chem FC3, Rancho Cordova, CA, USA) using chemiluminescent HRP substrate (Millipore, Burlington, MA, USA).

### 2.10. Antigen-Capture Enzyme-Linked Immunosorbent Assay (ELISA)

The TSP was isolated from 100 mg of young broccoli leaves utilizing extraction solution (1 M NaCl, 0.01% Triton X-100: pH 7.4, 5 mM CaCl_2_, 20 mM HEPES, 5 mM MgCl_2_, and phenylmethylsulfonyl fluoride at 1 mM). The polystyrene plates were used for performing the antigen-capture ELISA technique. Each well was first coated with 50 µL of monoclonal antibody (MAB 1.3.5.15 (Ritti01), obtained from German Cancer Research Centre (DKFZ), Heidelberg, Germany), diluted (1:250) in a coating buffer. The plate was then incubated overnight at 4 °C for the efficient adherence of antibody to each respective well. The first antibody was disposed from the plate, and each well was washed with wash buffer three times. To reduce the non-specific background noise, each well was blocked using 100 µL of blocking buffer for an hour at 37 °C. Following one hour of blocking, the ELISA plate was inverted and tapped for the removal of blocking solution. Further, 50 µL of protein sample was pipetted into the wells and the plate was incubated at 37 °C for one hour. Serially diluted VLPs (12, 6, and 3 ng/μL) served as positive controls and were subsequently used to quantify the plant-expressed L1 protein. Following incubation, the wells were rinsed thrice with wash buffer, and 50 µL of the second antibody, i.e., rabbit serum polyclonal antibody (p4543: obtained from German Cancer Research Centre (DKFZ), Heidelberg, Germany), prepared in antibody dilution buffer (1:5000), was added to each well, and ELISA plates were incubated at 37 °C for another hour. Following this incubation, 50 µL of goat anti-rabbit peroxidase conjugated antibody (GARPO) diluted in 1.5% milk/Tween-20 was applied in all wells of the plate, which were subsequently rinsed thrice with wash buffer and incubated again for one hour at 37 °C. Later, each well was rinsed properly with wash buffer three times for 5 min. In the final step, 100 µL of 3,3′,5,5′-tetramethylbenzidine (TMB) substrate, specific to the HRP enzyme, was pipetted into the wells, and the plate was kept in darkness for 20 to 30 min at room temperature. Finally, the absorbances were recorded at 495 nm using a spectrophotometer.

### 2.11. Quantification of Transgenic Protein

ELISA was used to quantify the L1 protein in accordance with the Verma et al. (2008) [34] methodology. As previously mentioned, TSP extracted from the transgenic broccoli was quantified and consequently utilized to evaluate the transgenic protein (TP) as a percentage of the total soluble protein of the altered leaf material. The formula used for calculating the percentage of TSP is stated as follows:

$$\%\;TSP=\left(\frac{TP}{TSP}\right)\times 100$$

### 2.12. Immunogenicity Assay of LTB-L1 Expressing Transformed Broccoli in Mice

The preclinical animal trials were performed in the primate facility at Quaid-i-Azam University, Pakistan. The approval for animal trials, conducted in accordance with standard procedures and protocols, was taken from the bioethical committee of Quaid-i-Azam University. Eleven sets of around eight-week-old mice, five per group, were selected and received administration orally and subcutaneously. Mice were grouped as follows: group 1—normal group with no dose, group 2—oral delivery of PBS solution, group 3—subcutaneous delivery of PBS solution, group 4—oral delivery of TSP from wild-type broccoli, group 5—subcutaneous delivery of TSP from wild-type broccoli, group 6—oral delivery of purified VLPs, group 7—subcutaneous delivery of purified VLPs, group 8—oral delivery of TSP from transgenic broccoli of HPV-16, group 9—subcutaneous delivery of TSP from transgenic broccoli of HPV-16, group 10—oral delivery of TSP from transgenic broccoli of HPV-18, group 11—subcutaneous delivery of TSP from transgenic broccoli of HPV-18. The Verma et al. (2008) [34] formula was used to determine the dosage for oral and subcutaneous injections:

$$Amount\;of\;transgenic\;protein=\frac{TP\times V_{PBS}}{W_{TLM}\times 10^{6}}$$

*TP* = concentration of transgenic protein in ng per mL, *V_PBS_* = volume of PBS in millilitres, and *W_TLM_* = weight of transformed leaf material in grams.

Using the parameters from the Verma et al. (2008) [34] formula, the quantity of transgenic broccoli tissue required to deliver an antigen-equivalent dose of 10 µg of L1 protein was calculated based on the experimentally determined L1 accumulation levels (0.33–0.35% of total soluble protein). The calculated amount of plant material was then resuspended in an appropriate volume of PBS according to the route of administration. For oral immunization, the antigen-equivalent plant extract was resuspended in 500 µL of sterile PBS and administered orally. For subcutaneous immunization, the same antigen-equivalent amount of extract was resuspended in 100 µL of sterile PBS and delivered by subcutaneous injection.

Doses were administered to the animals on days 1, 7, 14, and 21. One week following the final dosage, they were put to death, and the final blood was collected via heart puncture. Sera were obtained by the centrifugation of blood at 4000 rpm for 15 min at 4 °C, and were immediately saved at −80 °C for further use.

The mice’s blood serum was isolated from each mouse group during clinical trials and was eventually examined using ELISA to confirm the presence of IgG antibody expressed in response to the administered L1 recombinant vaccine. Each well of the microtiter plate was coated with 5 μg of transgenic broccoli TSP and left at 4 °C for 24 h. After three rounds of washing with TBS-T buffer, the plate was blocked for an hour at 37 °C using TBS-TM blocking solution (TBS with 0.3% Tween-20 and 3% skim milk). Then, 50 μL of isolated test serum was loaded onto the coated wells for capturing vaccine-stimulated mouse IgG antibody. Next, 50 μL of HRP-conjugated goat anti-mouse IgG secondary antibody (1:10,000) was pipetted in each well and the plate was incubated for one hour at 37 °C, followed by rinsing with TBS-T three times. Each well acquired 100 μL of TMB substrate. The plate was kept in the dark for 20 to 30 min at room temperature, and 0.16 M H_2_SO_4_ (100 μL) was added to stop the reaction. Finally, the absorbances were recorded at 495 nm using a spectrophotometer (BioRad, Tokyo, Japan).

### 2.13. Statistical Analysis

Microsoft Excel was applied to carry out statistical analysis, including calculating standard deviations and generating graphs.

## 3. Results

### 3.1. Gene Sequences of HPV-16 and -18 L1

**Figure 2:**
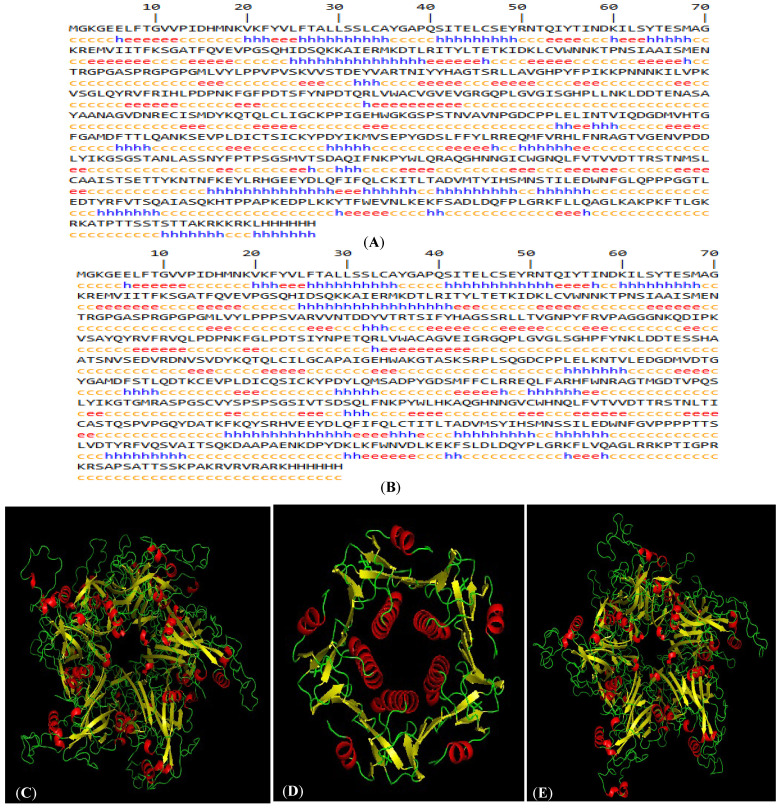

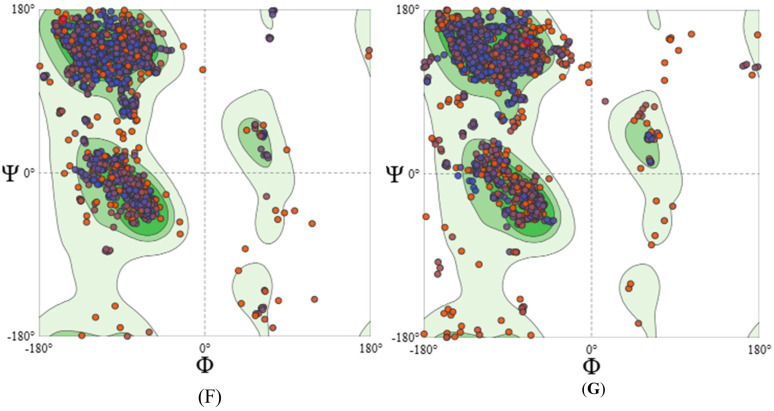
In silico characteristics of HPV-16 and HPV-18 LTB-L1 vaccine constructs. (**A**) HPV-16 LTB-L1 vaccine construct secondary structure, predicted by SOPMA protein secondary structure prediction server. (**B**) HPV-18 LTB-L1 vaccine construct secondary structure, predicted by SOPMA protein secondary structure prediction server. (**C**) Three-dimensional pentameric structure of HPV-16 L1 model (in HPV-16 LTB-L1 vaccine construct), predicted by SWISS-MODEL server. (**D**) Three-dimensional pentameric structure of LTB model (in HPV-16 LTB-L1 vaccine construct), predicted by SWISS-MODEL server. (**E**) Three-dimensional pentameric structure of HPV-18 L1 model (in HPV-16 LTB-L1 vaccine construct), predicted by SWISS-MODEL server. (**F**) Ramachandran plot of HPV-16 L1 model of HPV-16 LTB-L1 vaccine construct, predicted by SWISS-MODEL server. (**G**) Ramachandran plot of HPV-18 L1 model of HPV-18 LTB-L1 vaccine construct, predicted by SWISS-MODEL server.

The gene sequences of L1-WT16 and L1-WT18 (gene bank accession nos. KM058645.1 and MF288717.1, respectively) were retrieved and subsequently modified to enable the confinement of capsomeres rather than VLP formation. The HPV-16 LTB-L1 and HPV-18 LTB-L1 vaccine constructs were later converted to protein sequences by the Expasy Translate Tool for the downstream in silico analysis. The LTB-L1 vaccine construct sequences of HPV-16 and -18 contained 657 and 659 amino acids, respectively, as displayed in Figure 2A.

### 3.2. Epitope Prediction

The HPV-16 and HPV-18 LTB-L1 vaccine construct sequences were anatomized by different tools for epitope mapping to predict a number of epitopes that trigger the CTLs, HTLs and B-cells. The identified epitopes were screened for antigenicity, toxicity, and allergenicity by multiple bioinformatics tools to assess a set of protected epitopes, as provided in Table 1.

### 3.3. Physiochemical Properties and Characteristics Evaluation of Vaccine Constructs

Computational analysis showed that both HPV-16 and HPV-18 LTB-L1 vaccine constructs had favourable stability indices, molecular weights appropriate for L1-based antigens, and acceptable hydropathicity profiles. BLASTP comparison with the human proteome revealed no significant homology, which means that there was no probability of cross-reactivity.

Physicochemical property prediction indicated that both vaccine constructs may exhibit reduced solubility upon overexpression. The HPV-16 LTB-L1 vaccine construct was predicted to have reduced solubility upon overexpression with a probability score of 0.752058, while the HPV-18 LTB-L1 vaccine construct showed a predicted reduced insolubility probability of 0.672943. These predictions reflect theoretical behaviour under high-expression conditions. The predicted physicochemical properties are summarized in Table 2.

### 3.4. Validation of 3D Structures of Vaccine Constructs

Structural models were generated by modelling the HPV-16 LTB-L1 vaccine construct and HPV-18 LTB-L1 vaccine construct using template-based homology modelling. SWISS-MODEL generated independent models for LTB and L1 regions, rather than a fusion model of LTB-L1, due to the absence of an experimentally resolved template containing both LTB and L1 domains in a single fusion protein. SWISS-MODEL generated four structures for both HPV-16 and 18 LTB-L1 vaccine constructs; among these, two models were for LTB and the other two were for HPV-16/-18 L1. The 14-amino-acid linker region did not align with any structural template and therefore was not confidently modelled.

Among the determined models, the model with the highest sequence coverage and QMEAN Z-score was selected. In 3D structural models, L1 retained the characteristic β-jelly-roll fold and pentameric capsomeric arrangement. Similarly, the LTB adopted its canonical pentameric assembly. The detailed pentameric structural models of L1 of HPV-16 and HPV-18 LTB-L1 vaccine constructs were found to have acceptable stereochemical quality. Ramachandran plot analysis indicated that more than 90% of the residues were in the favoured regions, and ERRAT quality factors also indicated the accuracy of the predicted structures (Figure 2).

The best HPV-16 L1 model (in LTB-L1 construct) showed 98.54 sequence identity coverage and a QMEAN Z-score of −2.50, while 98.59% sequence identity coverage and −2.81 QMEAN Z-score were shown by the best HPV-18 L1 model (in LTB-L1 construct). The ERRAT quality factors of vaccine constructs were 81.2381 and 82.218 for HPV-16 and -18 L1 models, respectively. 3D pentameric structures of HPV-16 L1, LTB and HPV-18 L1 are shown in Figure 2C–E, respectively. Moreover, the Ramachandran plot of the HPV-16 L1 model showed 94.04% of residues in most favoured and 0.93% in unfavourable regions, while the Ramachandran plot of the HPV-18 L1 model showed 91.61% residues in most favoured and 2.56% in unfavourable regions, as shown in Figure 2F,G.

### 3.5. Discontinuous Mapping of B-Cell Epitopes

**Figure 3:**
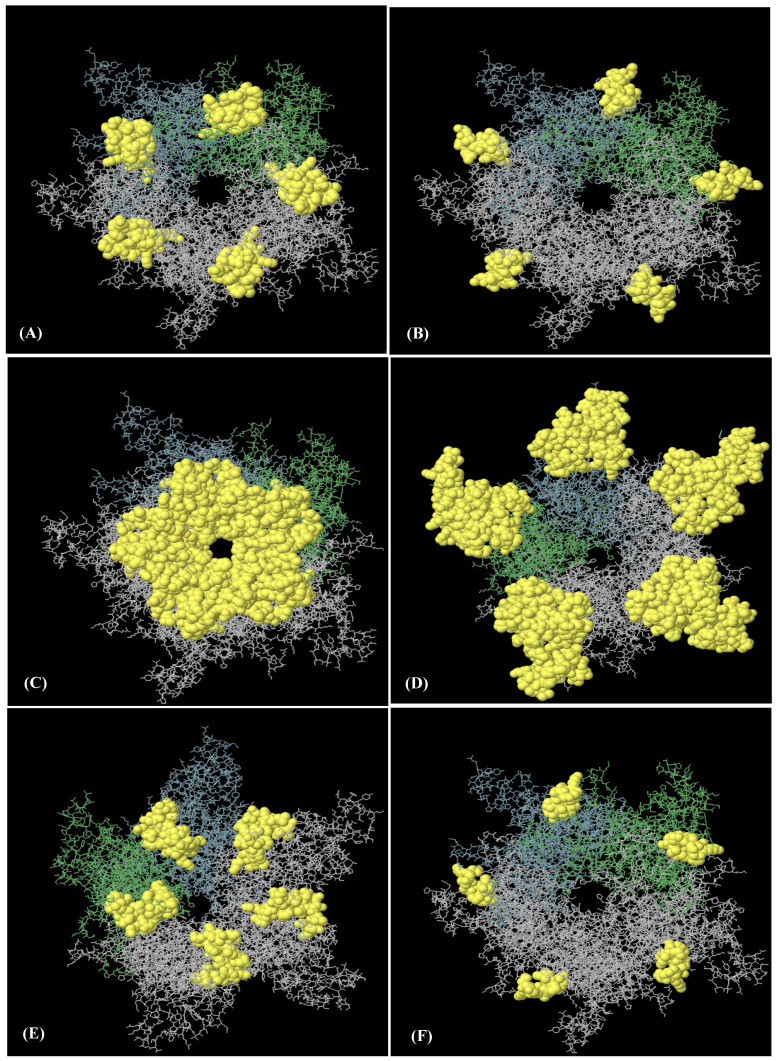

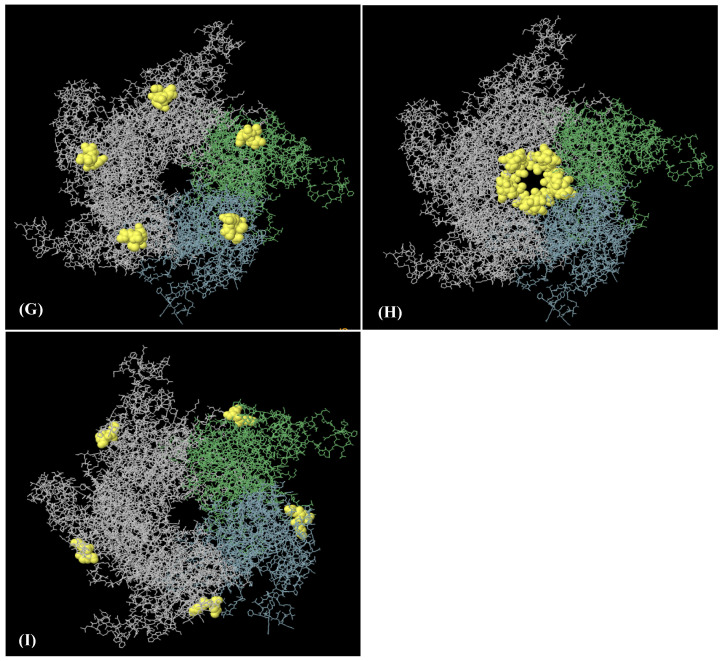
**Nine discontinuous B-cell epitopes mapped on HPV-16 L1 model of HPV-16 LTB-L1 vaccine constructs.** (**A**–**I**) A total of nine three-dimensional (3D) discontinuous B-cell epitopes were predicted using the ElliPro tool based on the HPV-16 L1 model. The discontinuous epitopes are shown in yellow on the pentameric vaccine. The residues comprising each predicted epitope are listed as follows: (**A**) residues 491–509, (**B**) residues 318–333, (**C**) residues 268, 270–292, 364, 402–406, 408, 409–437, (**D**) residues 181, 182, 458, 601–620, (**E**) residues 166–176, 225-244, 388, 465, 526–539, 541, 542, 545, 546, 548, 550–564, 566–584, 588, (**F**) residues 197–208, (**G**) residues 177–180, 524, (**H**) residues 443–451, and (**I**) residues 598–600.

**Figure 4:**
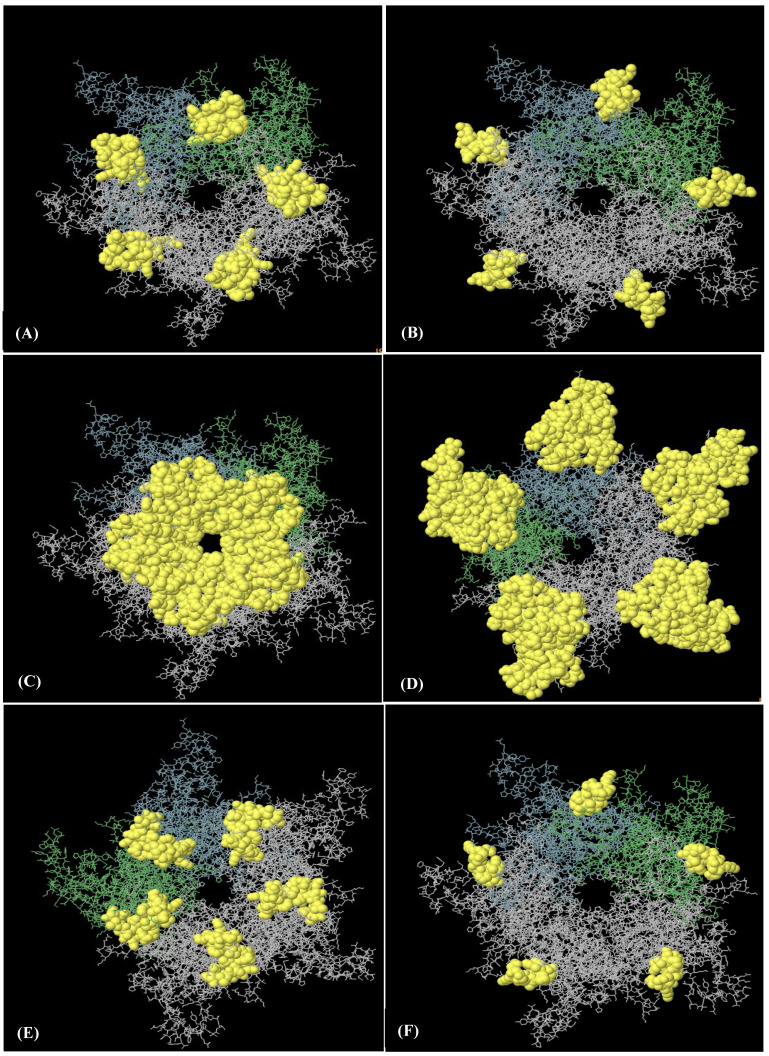
**Six discontinuous B-cell epitopes mapped on HPV-18 L1 model of HPV-18 LTB-L1 vaccine constructs.** (**A**–**F**) A total of eight three-dimensional (3D) discontinuous B-cell epitopes were predicted using the ElliPro tool based on the HPV-18 LTB-L1 vaccine model. The discontinuous epitopes are shown in yellow on the pentameric vaccine construct. The residues names comprising each predicted epitope are listed as follows: (**A**) residues 487–510, (**B**) residues 318–333, (**C**) residues 268, 270–292, 364, 402, 403–406, 408–437, 440, 442–451, (**D**) residues 167–169, 171–180, 225, 227–244, 388, 464, 465, 524, 525, 527–540, 542–544, 546, 547, 549–585 (**E**) residues 181, 455–458, 602–621, (**F**) residues 197–209.

An analysis of the discontinuous B-cell epitopes revealed nine conformational epitopes in the HPV-16 L1 model (in LTB-L1 construct) and six conformational epitopes in the HPV-18 L1 model (in LTB-L1 construct), suggesting the presence of multiple antibody-accessible sites (Figure 3 and Figure 4).

### 3.6. Codon Optimization and Final Transformation Vector

The GC contents of the optimized HPV-16 LTB-L1 and HPV-18 LTB-L1 vaccine constructs were 43.52% and 44.85%, respectively, which are in accordance with the codon usage of *Brassica oleracea.*

**Figure 5:**
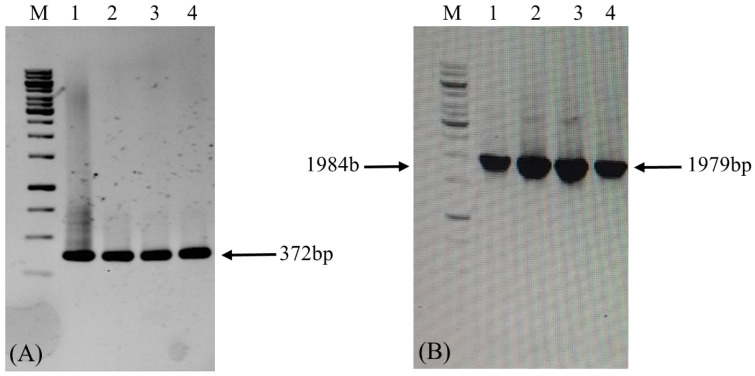
**Confirmation of *LTB-L1* gene integration in GV3101 strain of *Agrobacterium* by conventional PCR.** M: 1 kb DNA ladder; Lanes 1, 2: Validation of *Agrobacterium* GV3101 strain transformation with pEXP-HPV-16 binary vector; Lanes 3, 4: Validation of *Agrobacterium* GV3101 strain transformation with pEXP-HPV-18 binary vector. (**A**) The agarose gel image confirming PCR-based amplification of target *LTB-L1* gene in transformed *Agrobacterium* using primer pair 1. (**B**) The agarose gel image confirming PCR-based amplification of target *LTB-L1* gene in transformed *Agrobacterium* using primer pair 2.

The constructs were successfully assembled into the binary expression vectors pEXP-HPV-16 and pEXP-HPV-18 (Figure 1). PCR analysis confirmed the correct integration of the *L1* gene into the final expression vectors maintained in *Agrobacterium tumefaciens* strain GV3101 (Figure 5).

### 3.7. Regeneration of Transformed Broccoli Plants

**Figure 6:**
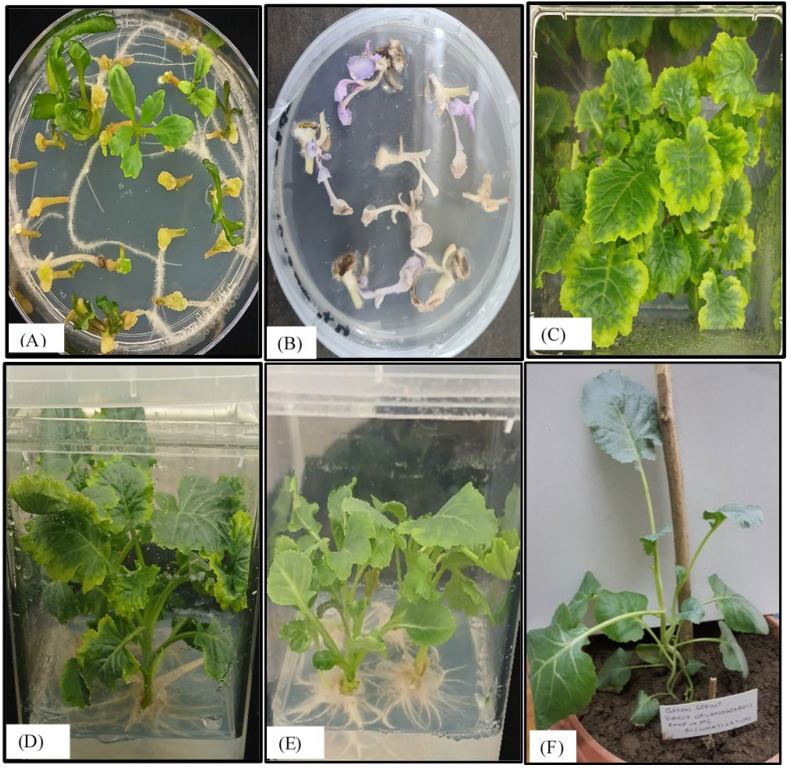
**Examination of in vitro regeneration of *Brassica oleracea* explants transformed with *LTB-L1* gene.** (**A**) Shoot regeneration of wild-type explants of *Brassica oleracea* on RMOP medium. (**B**) Untransformed explants of *Brassica oleracea* failing to survive on RMOP selection medium. (**C**) Shoot regeneration from transformed explants of *Brassica oleracea* supplemented with 75 mg/L kanamycin (above view). (**D**) Shoot regeneration from transformed explants of *Brassica oleracea* (side view). (**E**) Root emergence from the transgenic shoots of *Brassica oleracea*. (**F**) Acclimatization of regenerated transformed *Brassica oleracea*.

After transformation, the appearance of regenerated shoots was noticed only in explants transformed with pEXP-HPV-16 and pEXP-HPV-18, which appeared 7–10 days after selection. The untransformed *Brassica oleracea* explants were unable to survive on the selection medium (Figure 6). The regenerated shoots were successfully grown in soil after acclimatization. The transformed nature of the regenerated plants was confirmed by PCR analysis. Transformed broccoli plants appeared normally, like wild-type broccoli plants.

### 3.8. Confirmation of LTB-L1 Gene Within Broccoli Nuclear Genome

**Figure 7:**
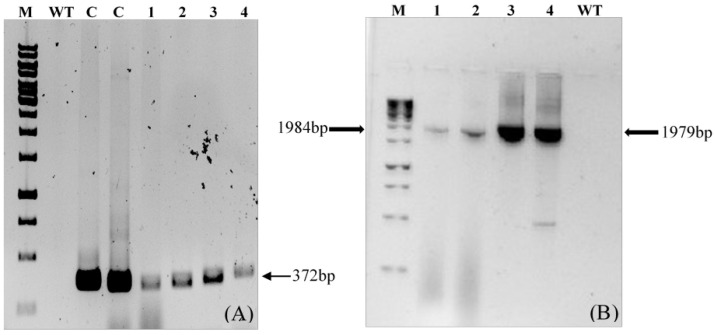
**Confirmation of *LTB-L1* gene integration in *Brassica oleracea* by conventional PCR.** M: 1kb DNA ladder; WT: Genomic DNA extracted from wild-type *Brassica oleracea* as negative control; C: Positive control; Lanes 1, 2: Two independent *Brassica oleracea* transgenic lines with HPV-16 *LTB-L1* gene; Lanes 3, 4: Two independent *Brassica oleracea* transgenic lines with HPV-18 *LTB-L1* gene. (**A**) The agarose gel image confirming PCR-based amplification of target *LTB-L1* gene in transformed *Brassica oleracea* using primer pair 1. (**B**) The agarose gel image confirming PCR-based amplification of target *LTB-L1* gene in transformed *Brassica oleracea* using primer pair 2.

PCR amplification of the transgene *LTB-L1* in regenerated broccoli plants confirmed the presence of the transgene, whereas there was no amplification in wild-type plants (Figure 7).

The transgenic broccoli plants that were positive for PCR were subjected to quantitative real-time PCR analysis to measure the copy number of the integrated *LTB-L1* gene relative to the endogenous β-actin gene. Standard curve analysis indicated acceptable linearity for the HPV-16 *LTB-L1*, HPV-18 *LTB-L1*, and β-actin genes, with correlation coefficients ranging from 0.89 to 0.92. Relative quantification indicated that two copies of the *LTB-L1* transgene were integrated into the broccoli genome for both HPV-16 and HPV-18.

### 3.9. Confirmation of LTB-L1 Expression in Transformed Plants by Western Blotting

**Figure 8:**
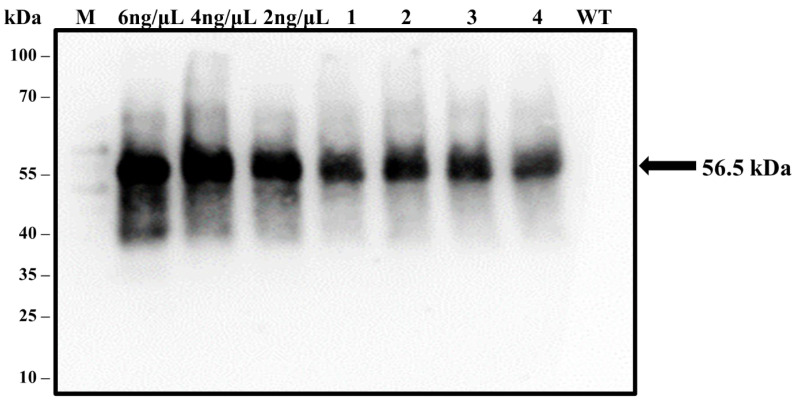
**Validation of LTB-L1 recombinant protein expression in transformed *Brassica oleracea* through western blotting.** M: 100 kDa protein ladder; 6, 4, 2 ng/µL: Varying concentrations of purified VLPs (baculovirus-derived) as positive controls; WT: TSP isolated from wild-type *Brassica oleracea* as negative control; Lanes 1, 2: Two independent *Brassica oleracea* transgenic lines with HPV-16_ LTB-L1 recombinant protein; Lanes 3, 4: Two independent *Brassica oleracea* transgenic lines with HPV-18 LTB-L1 recombinant protein. The presence of expressed LTB-L1 protein among TSP of transgenic plants was detected by the generation of bands on blotted membrane, followed by the treatment with L1-specific MD2H11 primary antibody and HRP-conjugated secondary antibody.

Western blotting analysis also confirmed the expression of the LTB-L1 recombinant protein in transgenic *Brassica oleracea* plants transformed with HPV-16 and HPV-18 constructs. Based on the predicted molecular weight of the full-length LTB-L1 fusion protein, bands of approximately 73 kDa were expected. However, immunoblot analysis consistently showed a single immunoreactive band of approximately 56.5 kDa in all transgenic lines (Figure 8), which corresponded to the molecular weight of the L1 monomer of both HPV-16 and HPV-18. No immunoreactive band was observed in wild-type plant extracts. The lack of a higher-molecular-weight band and the consistent appearance of the L1-sized product indicates the cleavage or post-translational separation of the LTB moiety from L1 in the plant expression system.

### 3.10. Antigen-Capture ELISA

**Figure 9:**
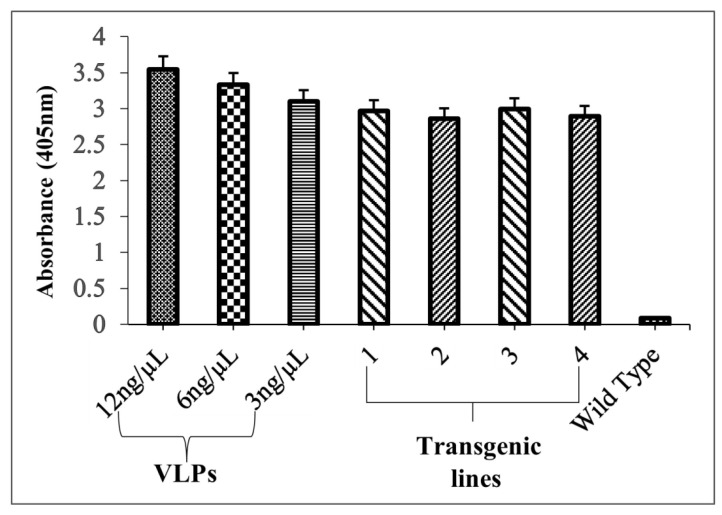
Quantification of correctly folded L1 recombinant protein in transformed *Brassica oleracea* via antigen-capture ELISA. VLPs: 12, 6, 3 ng/µL concentrations of purified VLPs (baculovirus-derived) as positive controls; Lanes 1, 2: Two independent *Brassica oleracea* transgenic lines with HPV-16 LTB-L1 recombinant protein; Lanes 3, 4: Two independent *Brassica oleracea* transgenic lines with HPV-18 LTB-L1 recombinant protein; WT: TSP isolated from wild-type *Brassica oleracea* as negative control. Three replicates were used for each.

Antigen-capture ELISA showed the existence of properly folded L1 protein (expressed as LTB-L1 fusion) in transgenic *Brassica oleracea*. ELISA signals in total soluble protein extracts from two independent transgenic lines exhibited strong signals, comparable to purified L1 VLP controls, indicating that conformational epitopes of capsomeric L1 had been retained and are effective in binding to conformational-specific antibody. Contrarily, wild-type plants did not show any signal in extracts (Figure 9). The strong signals shown by L1 capsomeres in antigen-capture ELISA support the epitope predictions made by the immunoinformatic modelling of L1, which suggests that the predicted conformational B-cell epitopes did not undergo any structural changes after the plant expression and processing. This correlation of in silico epitope mapping and experimental validation of ELISA substantiates the predictive validity of the computational vaccine design methods.

### 3.11. Quantification of Transgenic Protein via ELISA

Quantitative ELISA analysis revealed that the maximum accumulation levels of the plant-derived L1 protein (expressed as LTB-L1 fusion protein) corresponded to 0.33% and 0.35% of total soluble protein for the HPV-16 and HPV-18 constructs, respectively.

### 3.12. LTB-L1-Expressing Transgenic Broccoli Stimulated Immunogenic Responses in Mice

**Figure 10:**
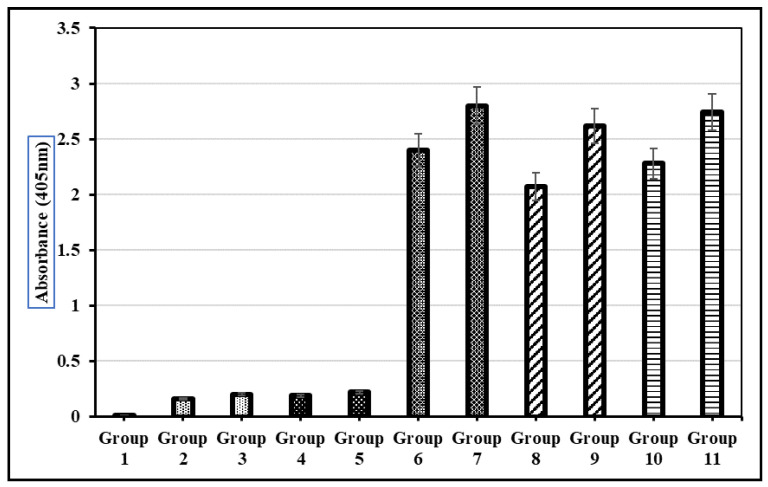
**Detection of L1 recombinant vaccine-stimulated IgG antibody in the blood sera of mice during preclinical trials.** Group 1: Normal group with no dose; Group 2: Oral delivery of PBS solution; Group 3: Subcutaneous delivery of PBS solution; Group 4: Oral delivery of TSP from wild-type *Brassica oleracea*; Group 5: Subcutaneous delivery of TSP from wild-type *Brassica oleracea*; Group 6: Oral delivery of purified VLPs; Group 7: Subcutaneous delivery of purified VLPs; Group 8: Oral delivery of TSP from transgenic *Brassica oleracea* with HPV-16; Group 9: Subcutaneous delivery of TSP from transgenic *Brassica oleracea* with HPV-16; Group 10: Oral delivery of TSP from transgenic *Brassica oleracea* with HPV-18; Group 11: Subcutaneous delivery of TSP from transgenic *Brassica oleracea* with HPV-18. Each mouse received an equivalent dose of 10 µg of transgenic L1 protein per immunization. Error bars indicate ± standard deviation (SD) (n = 5 mice per group).

Both the oral and subcutaneous administration of total soluble protein from LTB-L1-expressing transgenic broccoli induced anti-L1 IgG responses in mice. Mice that received transgenic plant extracts showed significantly higher antibody titres than those that received wild-type broccoli or PBS. The humoral immune responses triggered by plant-expressed HPV-16 and HPV-18 L1 capsomeres were comparable to those induced by baculovirus-derived purified L1 virus-like particles (Figure 10).

## 4. Discussion

The current study attempted to develop a cost-effective, plant-based vaccine candidate to combat HPV infection and cervical cancer by designing and expressing capsomeric pentamers of the HPV-16 and HPV-18 L1 proteins in *Brassica oleracea*. Cervical cancer continues to pose a major global health challenge, particularly affecting women in low- and middle-income countries, where limited access to screening programs and prophylactic vaccination contribute to high rates of morbidity and mortality [1,3,35,36]. The main etiological agent for the development of cervical cancer is the persistent infection with several high-risk types of human papillomaviruses (HPVs) [14]. However, most HPV infections are transient and are cleared by the host’s immune system. When the infection persists for several years, it can lead to the formation of cervical lesions and malignancy [37]. The risk of persistence and disease progression is also influenced by behavioural and socio-demographic factors such as poor menstrual hygiene, early marriage, unprotected sexual practices, and multiple sexual partners [38], which contribute to the high incidence of cervical cancer in low-resource countries. While existing HPV vaccines based on L1 virus-like particles (VLPs) are highly effective, their production mainly relies on expensive expression systems, cold-chain storage, and injection-based delivery, all of which limit their availability in resource-poor regions [39,40,41]. These constraints highlight the need for alternative vaccine platforms that are affordable, scalable, and suitable for oral delivery.

### 4.1. Capsomer-Based HPV Vaccines as Alternatives to VLPs

The traditional HPV vaccines are self-assembled L1 VLPs, which closely resemble the shape of the original virus and are capable of triggering effective neutralizing antibody responses [39,40]. Nevertheless, VLPs have structural complexity and thermolabile properties, which necessitate the preservation of cold-chain conditions [10]. Conversely, the smaller and more stable antigenic format is provided by L1 capsomeres, which are the pentameric subunits of the viral capsid. These capsomeres contain the major conformational epitopes required to stimulate antibody binding and have been reported to generate protective immune responses [42,43,44,45].

The current research was specifically aimed at the capsomeric assembly form of L1 rather than full virus-like particles (VLPs). Capsomeres are the pentameric subunits of the HPV capsid; they contain the important conformational epitopes required for antibody recognition and thus can serve as alternative vaccine antigens. Previous plant-based studies have shown that expressed HPV-16 L1 can assemble into capsomeres while retaining antigenicity, indicating their potential for next-generation vaccines [42].

In this study, specific N-terminal deletions and cysteine-to-serine substitutions were introduced into the L1 sequence to promote the formation of capsomeric structures rather than complete VLPs, following established protocols [42]. Although VLPs typically induce the most-neutralizing antibody titres, capsomeres have been reported to generate significant humoral responses and protective immunity in animal models, particularly when appropriate adjuvants and delivery strategies are employed [44,45]. Production-wise, capsomeres offer practical advantages, including structural simplicity, reduced assembly requirements, and improved suitability for cost-effective plant-based expression systems. The efficient accumulation of HPV L1 capsomeres in transplastomic plants further highlights their potential for scalable vaccine manufacturing [42]. Consistent with these reports, our results demonstrate that plant-produced L1 capsomeres maintained conformational epitopes and triggered robust humoral immune responses in mice, thus strengthening capsomeric L1 as a viable and economical alternative to VLP-based HPV vaccines.

### 4.2. In Silico Analysis of Vaccine Constructs

The in silico analysis of vaccine constructs has become a valuable tool for antigen selection and optimization prior to laboratory validation [20]. In this work, immunoinformatic methods were employed to assess antigenicity, allergenicity, toxicity, and epitope distribution within the LTB-L1 vaccine constructs. In line with earlier studies recognizing L1 as the most immunogenic HPV late protein [16,17], our analyses identified multiple linear and conformational B-cell epitopes across the L1 structure.

Physicochemical property predictions contributed to the computational assessment of the vaccine constructs. Solubility analysis predicted that both HPV-16 and HPV-18 LTB-L1 vaccine constructs might exhibit reduced solubility upon overexpression, with insolubility probabilities of 0.752058 for HPV-16 and 0.672943 for HPV-18. These findings are consistent with previous reports that the high-level expression of multimeric viral proteins often results in solubility challenges due to aggregation tendencies. Nonetheless, these predictions represent theoretical estimations under overexpression conditions and do not necessarily predict behaviour in biological systems such as plants, where protein folding, post-translational processing, and intracellular conditions can substantially influence solubility.

The intrinsic limits of the structural modelling of multidomain fusion proteins, such as HPV-16 and -18 LTB-L1 vaccine constructs, need acknowledgement. SWISS-MODEL and other homology-based servers are examples of template-based modelling techniques that depend on the availability of experimentally determined structures with a high degree of sequence similarity. There is currently no structure for an LTB-L1 fusion protein that has been determined experimentally. The modelling servers found different structural templates for each domain instead of a continuous fusion template since HPV L1 and LTB are unique structural domains with different oligomerization characteristics and folding patterns.

Furthermore, the modelling confidence is further reduced by the existence of a flexible 14-amino-acid linker between LTB and L1. Flexible linkers are frequently poorly resolved in crystallographic templates and lack a clear secondary structure. Therefore, such regions may be excluded from dependable structural predictions or modelled with poor confidence by template-based methods. In structural bioinformatics, these restrictions are widely known, especially when modelling proteins with flexible linking sections or multidomain fusion complexes. Therefore, rather than being a final depiction of the entire fusion architecture, the structural predictions made in this work were evaluated at the specific domain level (LTB and L1 separately). The main purpose of the modelling results was to bolster generic structural plausibility and epitope localization, not to assert particular interdomain arrangements or cooperative oligomerization behaviour.

Following structural evaluation, additional in silico investigations were carried out to maximize the vaccine construct expression potential. Codon usage bias, GC content, and mRNA stability are recognized as key determinants of heterologous gene expression in plants [26,27,46,47]. The optimized HPV-16 and HPV-18 LTB-L1 vaccine constructs exhibited GC contents of 43.52% and 44.85%, respectively, values compatible with the host genome and considered favourable for stable expression. These predictions aligned with the experimentally observed accumulation of L1 protein, highlighting the importance of codon optimization in linking computational design with successful biological expression.

Overall, the in silico analyses provided a robust framework to guide construct design and experimental validation. While computational methods have inherent limitations, their integration with experimental expression and immunogenicity data enhances the comprehensive evaluation of HPV L1 capsomer-based vaccine candidates.

### 4.3. Expression of LTB-L1 in Broccoli and Transgene Stability

*Agrobacterium*-mediated transformation facilitated the stable integration of the *LTB-L1* transgene into the broccoli nuclear genome, with qRT-PCR confirming two transgene copies for both HPV-16 and HPV-18 vaccine constructs. This copy number is typical for *Agrobacterium*-mediated transformations and generally promotes consistent expression while minimizing transgene silencing [33]. Successful plant regeneration and the molecular validation of transgenic lines further confirmed the feasibility of using *Brassica oleracea* as a production host for recombinant vaccine antigens. Broccoli was chosen as the host species due to its high biomass yield, well-established regeneration protocols, and potential for oral vaccine delivery [25]. Plant-based systems offer several advantages, including scalability, cost-effectiveness, and the ability to perform post-translational modifications, as required for proper antigen folding [27,48].

### 4.4. LTB-L1 Fusion and Evidence of LTB Cleavage in Plants

To enhance immunogenicity, the *L1* antigen was expressed as a translational fusion with the B subunit of *Escherichia coli* heat-labile enterotoxin (LTB), a known mucosal adjuvant that promotes antigen uptake and immune activation [27,28]. LTB is commonly used in plant-based vaccines designed for oral immunization because of its affinity for GM1 ganglioside receptors on mucosal surfaces.

Interestingly, it was found in western blot analysis that the major immunoreactive band pertained to the L1 monomer (~56.5 kDa) but not to the full-length LTB-L1 fusion (~73 kDa) protein, which appeared to be cleaved in the plant. Since this cleavage was not specifically targeted with the aid of a particular protease recognition site, it could be due to the endogenous proteolytic processing of the plant expression system. Recombinant fusion proteins have also been similarly processed in plant hosts and are typically linked with those proteins that are subject to protease action in the secretory pathway or during protein maturation [26,27]. Notably, it has been previously demonstrated that the partial or full cleavage of carrier domains in plant-expressed fusion proteins does not always affect the immunogenicity of antigens [28,49]. In line with these findings, antigen-capture ELISA and immunogenicity assays in the current study have shown that L1 epitopes were retained in their native form and were immunogenic. Thus, even though the fusion architecture was disturbed by the separation of LTB and L1, the immunologically significant L1 capsomere structural conformation did not seem to be disrupted.

### 4.5. Immunogenicity and Oral Vaccine Potential

Immunization trials demonstrated that both the oral and subcutaneous delivery of LTB-L1-expressing transgenic broccoli extracts elicited significant anti-L1 IgG responses in mice, comparable to those induced by baculovirus-derived VLPs. These results confirm the immunogenic potential of plant-produced L1 capsomeres and highlight their suitability for oral vaccination.

Plant-based vaccines are particularly advantageous for oral delivery due to the bio-encapsulation of antigens within plant cell walls, which protects proteins from enzymatic degradation in the gastrointestinal tract and promotes release in the intestine [49]. Oral vaccination minimizes the need for medical personnel, sterile injections, and cold-chain storage, making it ideal for large-scale deployment in resource-limited regions.

L1 levels (in the LTB-L1 fusion protein) in broccoli leaves (0.33–0.35% of soluble protein) are comparable to a number of reports on plant-based vaccines and seem adequate to develop effective oral vaccines [25]. It has been previously demonstrated that nuclear transformation systems normally produce recombinant proteins, often in the range of 0.01 to 0.5% TSP—variations depend on the host plant and expression strategy [26,27,28]. Nevertheless, higher expression levels (as much as 1–2%) have been observed previously for L1 capsomeres and VLPs [42,50] with plastid-based expression in tobacco. Consequently, the levels of the expression that were attained in broccoli through nuclear transformation in the current study are within an effective range and indicate the appropriateness of this system in the development of proof-of-concept plant-derived HPV vaccine candidates.

## 5. Conclusions and Future Perspectives

In summary, it was shown in this study that capsomeric HPV-16 and HPV-18 L1 antigens could be designed, expressed, and immunologically validated in *Brassica oleracea*. The plant-based antigens retained their conformation and induced robust humoral immunity, suggesting their future role as economical plant-based HPV vaccines. Since broccoli is an agricultural crop that can be eaten, there are also opportunities for using this platform to develop oral vaccines. In the future, further work could focus on assessing the stability of antigens in the gastrointestinal passage to optimize biomass processing methods like lyophilization for enhancing the storage and dose consistency. More specifically, proper dosage standardization in relation to the plant tissue material might continue to be a challenge for edible vaccines and will need dose optimization. Altogether, the current study contributes to the progress of scalable, accessible, and possibly orally deliverable vaccines to combat HPV and cervical cancer.

## Figures and Tables

**Table 1:** Predicted MHC-I, MHC-II, and B-cell binding epitopes and their characteristics.

Epitope Binding Predictions							Antigenicity		Allergenicity	Toxicity	
HPV Types	Allele	Position	Length	Epitope Sequence	Peptide Score	Percentile Rank	Protective Score	Probability		Toxicity Score	Probability
**MHC-I Binding Epitopes**											
**HPV-16**	HLA B* 15:01	104–113	10	RMKDTLRITY	0.973352	0.01	1.2885	Antigenic	Non-Allergen	−1.63	Non-Toxic
	HLA B* 44:03	514–522	9	EEYDLQFIF	0.973715	0.01	1.7384			−0.36	
	HLA A* 11:01	499–507	9	TTYKNTNFK	0.952679	0.01	1.1362			−0.63	
	HLA B* 53:01	223–231	9	LPDPNKFGF	0.944037	0.01	0.5171			−0.67	
	HLA A* 24:02	394–402	9	FYLRREQMF	0.930103	0.02	1.1062			−1.33	
	HLA A* 02:03	206–214	9	ILVPKVSGL	0.918948	0.03	0.4006			−0.83	
	HLA B* 08:01	596–605	10	NLKEKFSADL	0.814824	0.03	0.5760			−0.86	
**HPV-18**	HLA B* 44:03	515–523	9	EEYDLQFIF	0.973715	0.01	1.7384	Antigenic	Non-Allergen	−0.36	Non-Toxic
	HLA B* 15:01	104–113	10	RMKDTLRITY	0.973352	0.01	1.2885			−0.67	
	HLA A* 68:01	558–566	9	TTSLVDTYR	0.961923	0.02	0.4094			−0.51	
	HLA A* 31:01	504–512	9	ATKFKQYSR	0.939233	0.01	0.8894			−0.51	
	HLA A* 68:01	189–197	9	LTVGNPYFR	0.933423	0.05	1.2652			−0.70	
	HLA B* 07:02	438–446	9	SPSPSGSIV	0.910967	0.04	0.4061			−1.20	
	HLA B* 35:01	580–588	9	APAENKDPY	0.882663	0.05	0.7730			−0.64	
**MHC-II Binding Epitopes**											
**HPV-16**	HLA-DRB1*01:01	199–213	15	AMDFTTLQANKSEVP	0.9864	0.01	0.6328	Antigenic	Non-Allergen	−1.12	Non-Toxic
	HLA-DRB3*02:02	123–137	15	NASAYAANAGVDNRE	0.9282	0.04	0.9199			−0.53	
	HLA-DRB1*01:01	457–471	15	GRKFLLQAGLKAKPK	0.9835	0.02	0.8115			−0.63	
	HLA-DRB1*11:01	469–483	15	KPKFTLGKRKATPTT	0.8318	0.75	0.7043			−0.81	
	HLA-DPA1*01:03	344–358	15	ETTYKNTNFKEYLRH	0.7351	0.37	0.4763			−0.64	
**HPV-18**	HLA-DRB1*03:01	134-148	15	DVRDNVSVDYKQTQL	0.9203	0.25	1.2862	Antigenic	Non-Allergen	−0.81	Non-Toxic
	HLA-DRB5*01:01	319–333	15	NQLFVTVVDTTRSTN	0.6677	0.39	0.7780			−0.86	
	HLA-DPA1*02:01	474–488	15	GPRKRSAPSATTSSK	0.2678	0.41	1.1551			−1.00	
	HLA-DRB3*01:01	388–402	15	NSSILEDWNFGVPPP	0.5285	0.82	0.7489			−0.93	
	HLA-DRB1*08:02	38–52	15	GNPYFRVPAGGGNKQ	0.6877	0.99	0.4884			−0.92	
**B-cell Epitopes**											
**HPV-16**	_	220	16	RIHLPDPNKFGFPDTS	0.92	_	0.5073	Antigenic	Non-Allergen	−0.49	Non-Toxic
	_	317	16	KGSPSTNVAVNPGDCP	0.91	_	0.5046			−0.54	
	_	571	16	AIASQKHTPPAPKEDP	0.90	_	0.8702			−0.44	
	_	350	16	GFGAMDFTTLQANKSE	0.83	_	1.1201			−0.96	
	_	476	16	FVTVVDTTRSTNMSLC	0.85	_	1.1357			−0.58	
	_	261	16	VGISGHPLLNKLDDTE	0.85	_	0.5867			−0.96	
**HPV-18**	_	624	16	KPTIGPRKRSAPSATT	0.93	_	1.5692	Antigenic	Non-Allergen	−1.15	Non-Toxic
	_	371	16	CQSICKYPDYLQMSAD	0.92	_	0.5860			−0.06	
	_	620	16	GLRRKPTIGPRKRSAP	0.90	_	1.8854			−1.22	
	_	467	16	NGVCWHNQLFVTVVDT	0.86	_	0.6519			−1.09	
	_	318	16	GTASKSRPLSQGDCPP	0.87	_	1.0008			−0.35	
	_	512	16	RHVEEYDLQFIFQLCT	0.87	_	0.4841			−0.50	

**Table 2:** HPV-16 and HPV-18 LTB-L1 vaccine construct physiochemical properties and characteristics.

Physiochemical Properties			
**Parameters**		**HPV-16 Results**	**HPV-18 Results**
Total no. of amino acids		657	659
Molecular weight		73,259.65	73,380.30
Theoretical isoelectric point (pI)		8.84	8.69
Negative charged residues (Asp + Glu)		60	63
Positively charged residues (Arg + Lys)		71	72
Formula		**C_3280_H_5100_N_882_O_965_S_29_**	**C_3258_H_5067_N_897_O_976_S_30_**
Total no. of atoms		10,256	10,228
Instability index		34.62	45.02
Aliphatic index		74.93	72.32
Grand average of hydropathicity		−0.368	−0.413
**HPV-16 Characteristics**			
**Antigenicity**		**Allergenicity**	**Toxicity**
**Protective score**	**Probability**	**Probability**	**Probability**
0.5087	Antigenic	Non-allergen	Non-toxic
**HPV-18 Characteristics**			
**Antigenicity**		**Allergenicity**	**Toxicity**
**Protective score**	**Probability**	**Probability**	**Probability**
0.5152	Antigenic	Non-allergen	Non-toxic

## Data Availability

The raw data supporting the conclusions of this article will be made available by the authors on request.
